# Combination of sodium-glucose cotransporter 2 inhibitor and dipeptidyl peptidase-4 inhibitor in type 2 diabetes: a systematic review with meta-analysis

**DOI:** 10.1038/s41598-018-22658-2

**Published:** 2018-03-13

**Authors:** Se Hee Min, Jeong-Hwa Yoon, Sun Joon Moon, Seokyung Hahn, Young Min Cho

**Affiliations:** 1 Division of Endocrinology and Metabolism, Department of Internal Medicine, Seoul National University College of Medicine, Seoul, South Korea; 2 Interdisciplinary Program in Medical Informatics, Seoul National University College of Medicine, Seoul, South Korea; 3 Department of Medicine, Seoul National University College of Medicine, Seoul, South Korea

## Abstract

Sodium glucose cotransporter 2 (SGLT2) inhibitors and dipeptidyl peptidase-4 (DPP4) inhibitors have complementary mode of action. For the meta-analysis comparing the efficacy and safety between SGLT2 inhibitor plus DPP4 inhibitor (SGLT2i/DPP4i) and placebo plus DPP4 inhibitor (PCB/DPP4i) in patients with type 2 diabetes mellitus (T2DM), we selected randomized controlled trials from electronic databases by predefined criteria. The primary outcome of interest was the change in glycated hemoglobin A1c (HbA1c) from baseline. Of 605 potentially relevant studies, 7 eligible RCTs comprising 2,082 patients were included.SGLT2i/DPP4i showed a greater reduction in HbA1c (weighted mean difference −0.6%, 95% CI −0.7 to −0.5%), fasting plasma glucose, 2 h postprandial plasma glucose, and body weight compared to PCB/DPP4i. The risk of hypoglycemia increased in SGLT2i/DPP4i compared to that in PCB/DPP4i only when insulin or sulfonylureas were included as a background therapy. The risk of urinary tract infection was not increased in SGLT2i/DPP4i; however, the risk of genital infection increased upon adding SGLT2 inhibitors to pre-existing DPP4 inhibitors. In conclusion, compared to PCB/DPP4i, SGLT2i/DPP4i achieved better glycemic control and greater weight reduction without increasing the risk of hypoglycemia and urinary tract infection in patients with inadequately controlled T2DM.

## Introduction

The pathogenesis of type 2 diabetes is intertwined with multiple different mechanisms, which encompasses decreased insulin secretion, decreased insulin sensitivity, increased hepatic glucose production, decreased responses to incretin hormones, and increased renal reabsorption of glucose^1^. Therefore, multiple strategies are often required to effectively control hyperglycemia in patients with type 2 diabetes. The combinatory use of different anti-diabetic agents with complementary mechanisms of action may enhance the glucose-lowering effect without compromising drug safety.

Newer anti-diabetic agents such as sodium glucose cotransporter 2 (SGLT2) inhibitors and dipeptidyl peptidase-4 (DPP4) inhibitors are very useful in that they rarely cause common adverse effects of other oral hypoglycemic agents, such as weight gain and hypoglycemia. SGLT2 inhibitors reduce hyperglycemia by increasing urinary glucose excretion independent of insulin secretion or action^2,3^. DPP4 inhibitors, which inhibit the breakdown of active incretin hormones, improve glucose homeostasis by increasing insulin secretion and decreasing glucagon secretion in a glucose-dependent manner^4,5^. In this regard, the combination of these two drugs could be effective and safe for the treatment of hyperglycemia in patients with suboptimally controlled type 2 diabetes. Therefore, we performed a systematic review and meta-analysis to assess the efficacy and safety of a combination of an SGLT2 inhibitor and a DPP4 inhibitor in patients with suboptimally controlled type 2 diabetes.

## Methods

Our systematic review and meta-analysis was performed using a pre-developed protocol that defined study selection criteria, search terms, resources to be searched, and data extraction and analysis strategy. The study was conducted and reported according to the Preferred Reporting Items for Systematic Reviews and Meta-Analysis (PRISMA) statement^6^.

### Eligibility criteria

We included randomized controlled trials (RCTs) that compared the SGLT2 inhibitor plus the DPP4 inhibitor (SGLT2i/DPP4i) with placebo plus the DPP4 inhibitor (PCB/DPP4i) in patients with type 2 diabetes concurrently treated with or without other anti-diabetic agents. The eligible RCTs were written in English, had at least 12 weeks of trial duration, and provided information of glycated hemoglobin A1c (HbA1c) changes from baseline. Studies with a duplication of data or an extended phase of the original ones were excluded. Two authors (S.H.M. and S.J.M.) independently evaluated study titles, abstracts, and full texts, with disagreements resolved by discussion or by a third investigator (Y.M.C.).

### Data sources and search strategies

We systematically searched to identify potentially eligible studies from inception to December 2016 from the following electronic databases: MEDLINE, EMBASE, and Cochrane Central Register of Controlled Trials (CENTRAL). We also searched ClinicalTrials.gov to find out unpublished studies. The following search terms were used: “DPP-4 inhibitor”, “vildagliptin”, “sitagliptin”, “linagliptin”, “alogliptin”, “saxagliptin”, “gemigliptin”, “dutogliptin”, “gosogliptin”, “anagliptin”, “tenegliptin”, “evogliptin”, “omarigliptin”, “trelagliptin”, “SGLT2 inhibitor”, “dapagliflozin”, “canagliflozin”, “empagliflozin”, “ipragliflozin”, “luseogliflozin”, “tofogliflozin”, “ertugliflozin”, “sotagliflozin”. Both generic names and pre-marketed names of DPP-4 inhibitors and SGLT2 inhibitors were included. The search results were limited to humans and clinical trials, which were adjusted to comply with the relevant rules in each database. The detailed search strategies are provided in Supplementary Text 1.

### Data extraction

Two authors (S.H.M. and J.-H.Y.) independently extracted data from the selected studies using a standardized form. Any discrepancies were discussed by all authors and resolved by the consensus. The primary efficacy outcome was the change in HbA1c from the baseline to the end of the trial, and the secondary efficacy outcomes included the change in fasting plasma glucose (FPG) levels, 2 h postprandial glucose (PPG), the proportion of patients achieving HbA1c below 7%, body weights, systolic blood pressure (SBP), and lipid profiles from baseline. The safety outcomes included the risk of hypoglycemia, urinary tract infection (UTI), and genital infection. The additional extracted information was as follows: the name of first author, year of publication, drug name and doses of SGLT2 inhibitors and DPP4 inhibitors, National Clinical Trial number, duration of treatment, concomitantly used anti-diabetic medications, number of randomized patients, age, percentage of men, body mass index (BMI), baseline values of HbA1c, and duration of diabetes. For the dose-range studies, the results with the maximum approved doses were selected for comparability among different drugs in a class.

For the continuous outcome values, the intergroup mean difference between the treatment and the placebo group and its standard error were extracted as the summary measure. We used the least squares (LS) mean differences from analysis of covariance (ANCOVA) between groups with adjustment for covariates if available. In some studies, we used the simple arithmetic mean difference for the outcomes, which were not adjusted. For the dichotomous outcomes, the number of patients with or without the outcome was extracted.

### Assessment of the study quality and risk of bias

Quality assessments were carried out by two independent reviewers (S.H.M. and J.-H.Y.) using the Cochrane Collaboration’s tool, and any controversies were resolved by mutual discussion. We considered the following aspects for the risk of bias: including adequacy of random sequence generation, allocation concealment, blinding, completeness of outcome data, selective outcome reporting, and other biases that could introduce confounding effects. For completeness of outcome data, analysis based on intention-to-treat analysis or full analysis set was considered as low risk. Selective reporting was assessed by comparing the reported outcomes with pre-specified study endpoints. Possibility for confounding biases was assessed by checking the comparability of participants’ baseline characteristics including age, sex, BMI, baseline values of HbA1c, and duration of diabetes between groups.

### Statistical analysis

Weighted mean difference (WMD) and 95% confidence intervals (CIs) between treatment groups were calculated for the change in HbA1c, FPG, 2 h PPG, body weight, SBP, and serum lipids from baseline. For dichotomous outcomes, including the proportion of patients achieving HbA1c < 7% and the risk of hypoglycemia, UTI, and genital infection, we calculated the relative risks (RRs) and their 95% CIs. Additionally, a sensitivity analysis was done for the risk of hypoglycemia by excluding a trial which permitted the concomitant use of anti-diabetic agents associated with increased risk of hypoglycemia, such as insulin or insulin secretagogues. A random effects model^7^ was used to account for heterogeneity across included studies for calculating pooled estimates and p-values, and an incorporated result was regarded as statistically significant if p-values < 0.05. The I^2^ statistic was calculated to evaluate the extent of heterogeneity across the studies. The potential risk of publication bias was investigated graphically by constructing funnel plot for the primary outcome along with assessment of funnel plot asymmetry by performing Egger’s test. In addition, we performed subgroup analyses to assess a difference in the effect depending on two modes of combination based on whether the subjects (i) used the SGLT2 inhibitor and DPP4 inhibitor simultaneously; or (ii) used the SGLT2 inhibitor sequentially after using the DPP4 inhibitor first. We then integrated the results of subgroup analyses for deriving the overall outcome. We used STATA (version 12; Stata Corp, College Station, TX, USA) for all the analyses.

### Data Availability

All data analysed during this study are included in this published article (and its Supplementary Information files).

## Results

### Search results and study characteristics

**Figure 1:**
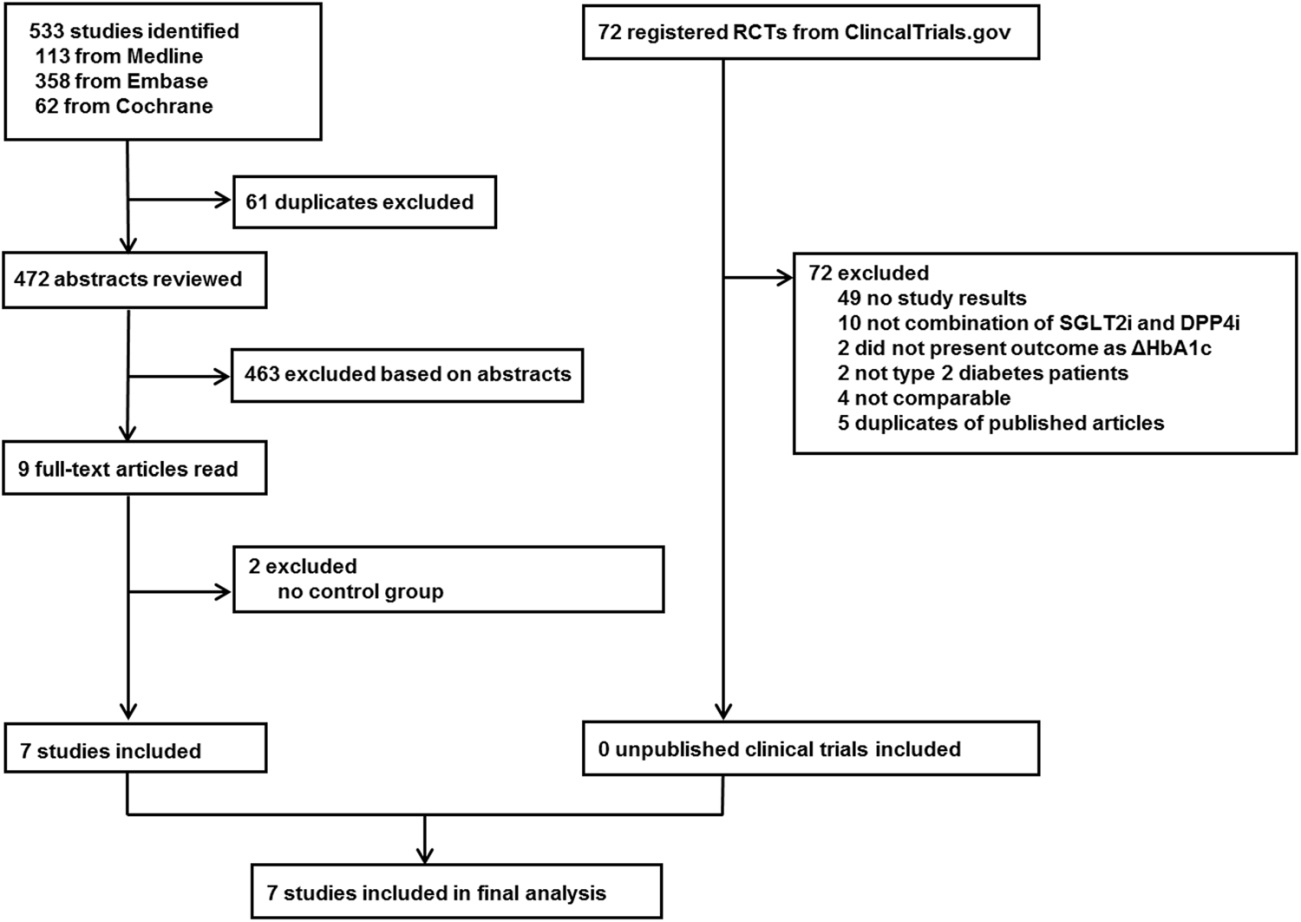
Study selection process. RCT = randomized controlled trial.

A total of 533 potentially relevant studies were retrieved from initial database search form MEDLINE, EMBASE, and CENTRAL, of which 7 articles met all inclusion criteria^8–14^. Among the 72 relevant search results from ClinicalTrials.gov, there was no additional clinical trial to be included in the analysis. Therefore, 7 studies involving 2,082 study participants (1,051 randomized to SGLT2i/DPP4i group and 1,031 randomized to PCB/DPP4i group) with a mean trial duration of 23 weeks (range 18 to 24 weeks) were finally enrolled in this study. The summary of study selection process is presented in Fig. 1 and the characteristics of included studies are provided in Table 1. Three out of seven studies compared the simultaneous combination of an SGLT2 inhibitor and a DPP4 inhibitor with the addition of a DPP4 inhibitor alone in drug-naïve or metformin failure patients, whereas the other four studies compared the addition of an SGLT2 inhibitor with a placebo as add-on therapy in patients inadequately controlled with a DPP4 inhibitor. All included studies, except one trial (initial combination of SGLT2 inhibitor and DPP4 inhibitor)^9^, permitted concurrent use of metformin. Of note, one trial did not restrict any background anti-diabetic agents and permitted concurrent use of insulin or insulin secretagogues^11^.

**Table 1:** Characteristics of the included studies in the meta-analysis.

Study Source	Study duration (weeks)	Study arms	Randomized participants, N	Mean age (years)	Men (%)	Baseline BMI (kg/m^2^)	Baseline HbA_1c_ (%)	Mean duration of diabetes (years)
**Simultaneous combination of SGLT2i and DPP4i in drug-naïve or metformin failure patients**								
DeFronzo 2015^8^	24	Empagliflozin 25 mg qd + linagliptin 5 mg qd + metformin	134	57.1	53.7	30.6	7.90	NR
		Placebo + linagliptin 5 mg qd + metformin	128	56.2	50.0	30.6	8.02	NR
Lewin 2015^9^	24	Empagliflozin 25 mg qd + linagliptin 5 mg qd	134	54.2	52.2	31.8	7.99	NR
		Placebo + linagliptin 5 mg qd	133	53.8	56.4	31.9	8.05	NR
Rosenstock 2015^10^	24	Dapagliflozin 10 mg qd + saxagliptin 5 mg qd + metformin	179	53.0	47.0	31.8	8.92	7.1
		Placebo + saxagliptin 5 mg qd + metformin	176	55.0	53.0	31.8	9.03	8.2
**Sequential combination by adding SGLT2i in patients uncontrolled with DPP4i**								
Fulcher 2016^11^	18	Canagliflozin 300 mg qd + DPP4 inhibitor ± insulin ± OADs^*^	111	62.7	73.0	32.3	8.00	13.2
		Placebo + DPP4 inhibitor ± insulin ± OADs^*^	102	63.9	59.0	32.3	8.10	12.5
Jabbour 2014^12^	24	Dapagliflozin 10 mg qd + sitagliptin 100 mg qd ± metformin	223	54.8	57.0	NR	7.90	5.7
		Placebo + sitagliptin 100 mg qd ± metformin	224	55.0	52.7	NR	8.00	5.6
Mathieu 2015^13^	24	Dapagliflozin 10 mg qd + saxagliptin 5 mg qd + metformin	160	55.2	43.8	31.2	8.24	7.2
		Placebo + saxagliptin 5 mg qd + metformin	160	55.0	47.5	32.2	8.17	8.0
Søfteland 2016^14^	24	Empagliflozin 25 mg qd + linagliptin 5 mg qd + metformin	110	55.4	64.5	29.9	7.97	NR
		Placebo + Linagliptin 5 mg qd + metformin	108	55.9	55.5	29.6	7.97	NR

Abbreviations: BMI, body mass index; N, number; NR, not recorded.

*Metformin, sulfonylurea, thiazolidinedione, alpha-glucosidase inhibitor, or other antihyperglycemic agent.

### Quality assessment of included studies

All seven RCTs were double-blinded and were therefore classified as low risk. For assessing the bias of incomplete outcome data and selective reporting, all studies were classified to be low risk. Five out of the seven studies did not state the process of random sequence generation^8,10,12–14^. The allocation concealment was performed appropriately in all included studies, and demographics and baseline characteristics of all studies were generally well balanced and therefore judged that studies were not prone to confounding biases. The summarized risk of bias is depicted in Supplementary Figure 1.

### Efficacy outcomes

**Figure 2:**
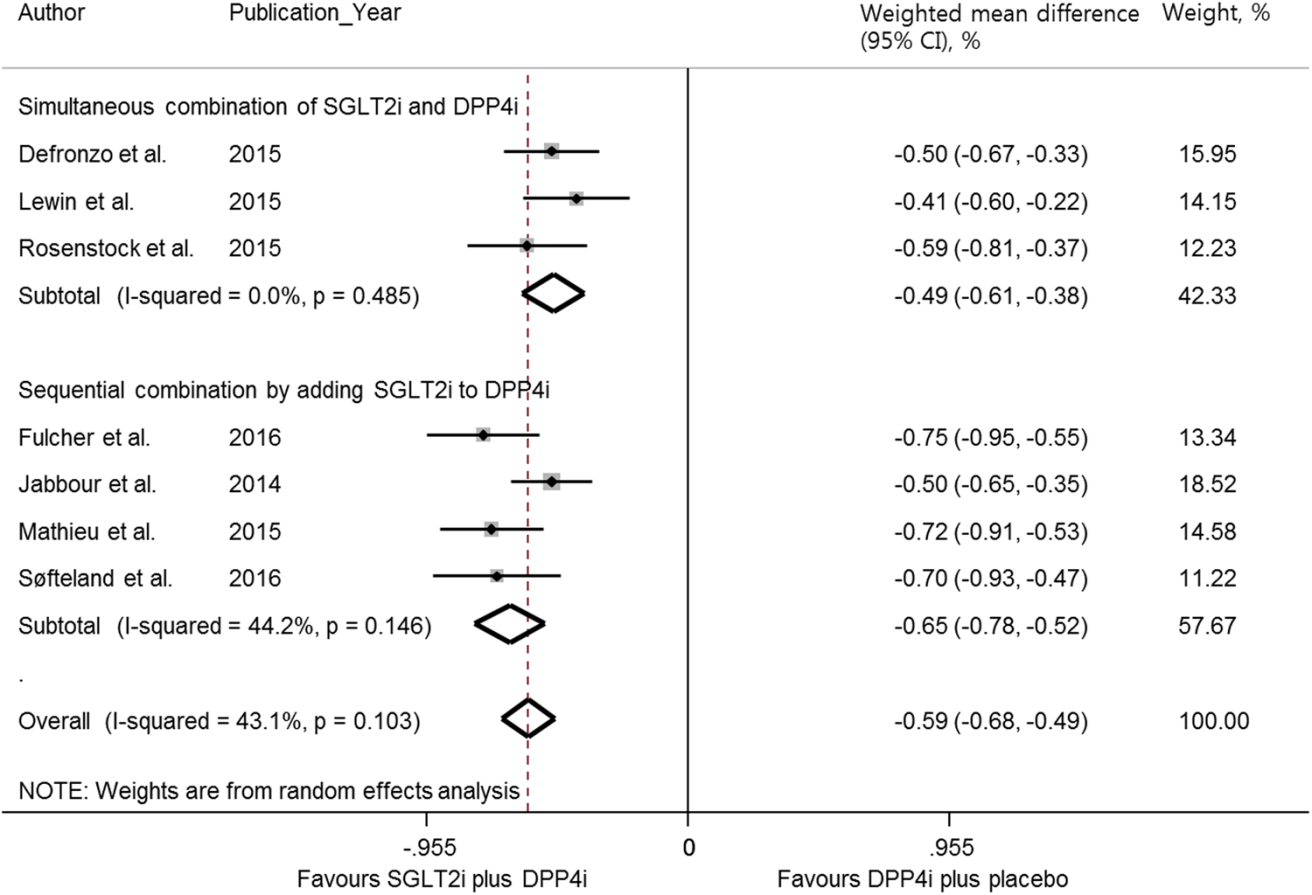
Meta-analysis for the primary efficacy outcome. Weighted mean difference in change in HbA1c (%) from baseline with SGLT2 inhibitor plus DPP4 inhibitor versus placebo plus DPP4 inhibitor with the subgroup analyses according to the simultaneous combination and sequential addition of two drugs. The squares represent an individual study’s effects, and the size of squares reflects the study’s weight, with the horizontal lines extending from the symbols representing 95% CIs. The diamonds indicate the pooled estimates. CIs = confidence intervals; PCB/DPP4i = placebo plus DPP4 inhibitor; SGLT2i/DPP4i = SGLT2 inhibitor plus DPP4 inhibitor.

Meta-analysis of the seven studies showed that SGLT2i/DPP4i led to greater improvement in HbA1c than PCB/DPP4i (WMD −0.59%, 95% CI −0.68 to −0.49%) (Fig. 2). Both simultaneous combination and sequential addition of SGLT2 inhibitors to DPP4 inhibitors showed a greater reduction in HbA1c than the respective PCB/DPP4i group (WMD −0.49%, 95% CI −0.61 to −0.38% and WMD −0.65, 95% CI −0.78 to −0.52%, respectively). There was a trend toward greater HbA1c reduction in sequential addition of SGLT2 inhibitors to DPP4 inhibitors compared with the simultaneous combination, although the difference was not statistically significant (p = 0.153).

**Figure 3:**
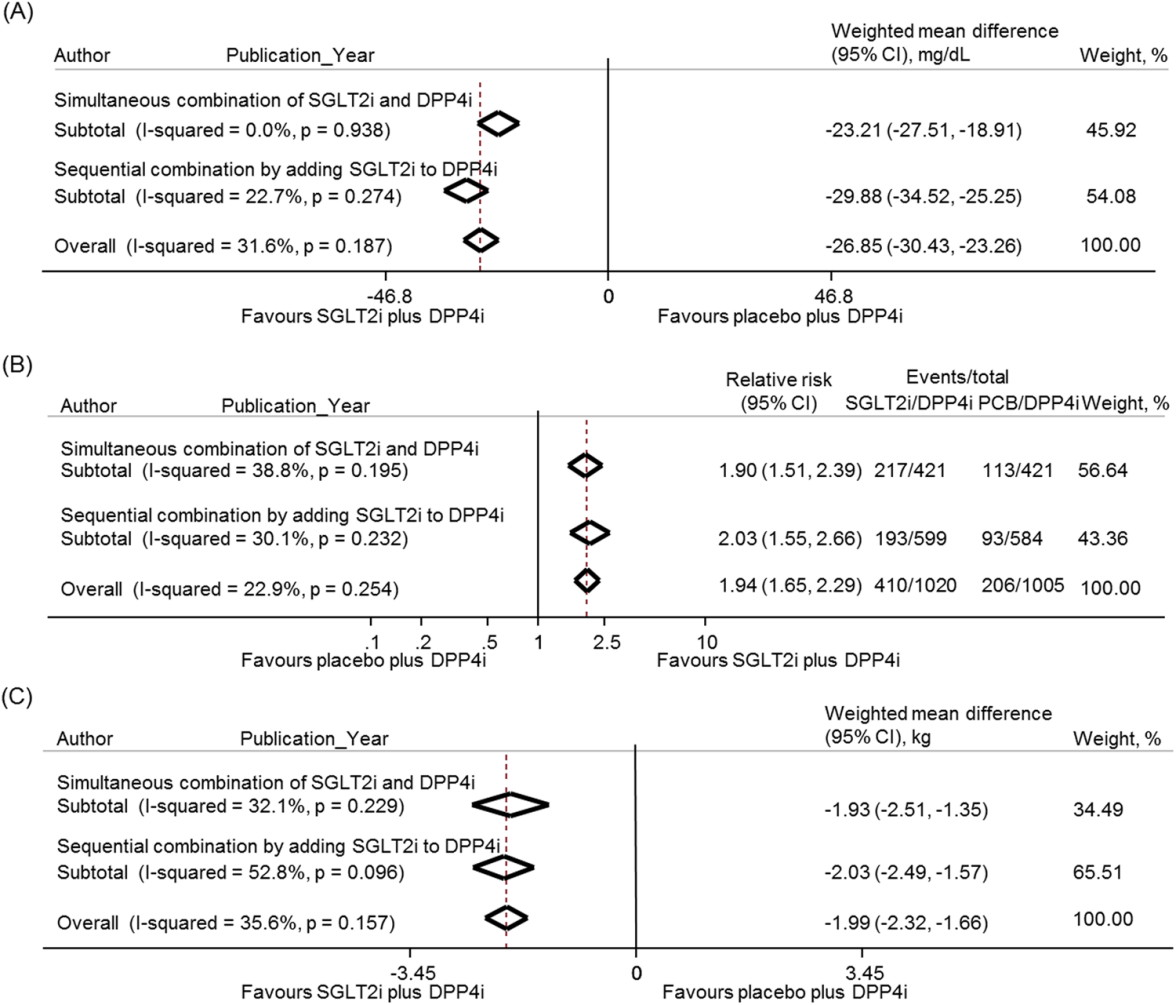
Meta-analysis for the secondary efficacy outcomes. (**A**) Weighted mean difference in change in fasting plasma glucose (mg/dL) from baseline with SGLT2 inhibitor plus DPP4 inhibitor versus placebo plus DPP4 inhibitor. (**B**) Relative risk of the proportion of participants achieving the target HbA1c level of <7.0% with SGLT2 inhibitor plus DPP4 inhibitor compared with placebo plus DPP4 inhibitor. (**C**) Weighted mean difference in change in body weight (kg) from baseline with SGLT2 inhibitor plus DPP4 inhibitor versus placebo plus DPP4 inhibitor. The subgroup analyses were presented separately according to the simultaneous combination and sequential addition of two drugs. The diamonds indicate the pooled estimates. PCB/DPP4i = placebo plus DPP4 inhibitor; SGLT2i/DPP4i = SGLT2 inhibitor plus DPP4 inhibitor.

All seven studies assessed the change in FPG from baseline (Fig. 3A). The SGLT2i/DPP4i group showed a greater reduction in FPG than the PCB/DPP4i group in the pooled analysis (WMD −26.9 mg/dL, 95% CI −30.4 to −23.3 mg/dL), and in the subgroups with either simultaneous or sequential mode of combination (WMD −23.2 mg/dL, 95% CI −27.5 to −18.9 mg/dL and WMD −29.9, 95% CI −34.5 to −25.3 mg/dL, respectively). There was also a trend toward a greater decrease in FPG in the sequential combination group compared with the simultaneous combination group (p = 0.081). In addition, only three studies reported the changes in 2 h PPG from baseline^10,12,13^ (Supplementary Figure 2) and the reduction of 2 h PPG was greater in the SGLT2i/DPP4i group than in the PCB/DPP4i group (WMD −41.4 mg/dL, 95% CI −47.0 to −35.7 mg/dL).

All seven studies assessed the proportion of patients attaining HbA1c levels of <7.0%^8–14^ (Fig. 3B). The RR for achieving target HbA1c was higher in the SGLT2i/DPP4i group compared with the PCB/DPP4i group (RR 1.9, 95% CI 1.7–2.3) and in the subgroups of the simultaneous combination group and the sequential combination group (RR 1.9, 95% CI 1.5–2.4 and RR 2.0, 95% CI 1.6–2.7, respectively).

For body weight change, all seven studies provided the results (Fig. 3C). Significantly greater body weight reduction was shown in the SGLT2i/DPP4i group compared with the PCB/DPP4i group (WMD −2.0 kg, 95% CI −2.3 to −1.7 kg) and in the subgroups of the simultaneous and sequential combination (WMD −1.9 kg, 95% CI −2.5 to −1.4 kg and WMD −2.0 kg, 95% CI −2.5 to −1.6 kg, respectively). The difference between the two subgroups was not statistically significant (p = 0.804).

Four studies reported a change in SBP^10,11,13,14^ (Supplementary Figure 3). A pooled analysis of four studies showed a 3.2 mmHg (95% CI −4.5 to −1.8 mmHg) reduction in SBP in the SGLT2i/DPP4i group compared with the PCB/DPP4i group. Serum lipids such as total cholesterol, triglycerides, LDL cholesterol (LDL-C), and HDL cholesterol (HDL-C) were assessed by the percent change from baseline^10–14^. In the pooled analysis, LDL-C and HDL-C were significantly increased in the SGLT2i/DPP4i group compared with the PCB/DPP4i group, whereas total cholesterol and triglycerides were not significantly different (Supplementary Figures 4–7). Subgroup analyses were not performed due to a paucity of data.

### Safety outcomes

**Figure 4:**
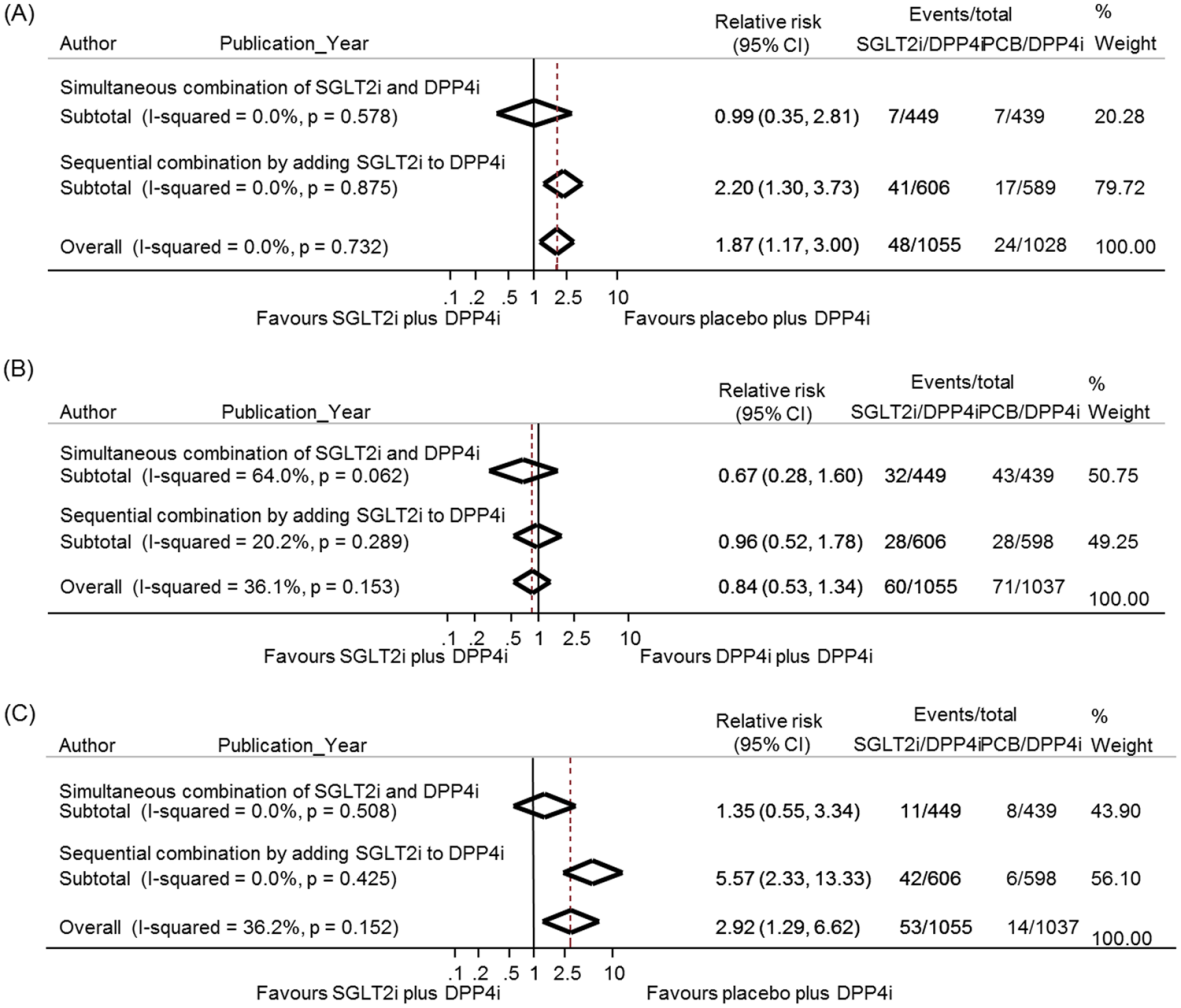
Meta-analysis for the safety outcomes. (**A**) Relative risks of hypoglycemia with SGLT2 inhibitor plus DPP4 inhibitor compared with placebo plus DPP4 inhibitor. (**B**) Relative risks of urinary tract infection with SGLT2 inhibitor plus DPP4 inhibitor compared with placebo plus DPP4 inhibitor. (**C**) Relative risks of genital infection with SGLT2 inhibitor plus DPP4 inhibitor compared with placebo plus DPP4 inhibitor. The subgroup analyses were presented separately according to the simultaneous combination and sequential addition of two drugs. The diamonds indicate the pooled estimates. PCB/DPP4i = placebo plus DPP4 inhibitor; SGLT2i/DPP4i = SGLT2 inhibitor plus DPP4 inhibitor.

All seven studies were available for the analysis of hypoglycemia^8,10–12,14^ (Fig. 4A). The definition of hypoglycemia was slightly different among studies (Supplementary Table S1). In the pooled analysis, the risk of hypoglycemia was higher in the SGLT2i/DPP4i group than in the PCB/DPP4i group (RR 1.9, 95% CI 1.2–3.0). The subgroup analyses showed that the sequential addition of an SGLT2 inhibitor to a DPP4 inhibitor increased the risk of hypoglycemia compared with the PCB/DPP4i group (RR 2.2, 95% CI 1.3–3.7), whereas the simultaneous combination of the SGLT2i/DPP4i group did not increase the risk of hypoglycemia (RR 1.0, 95% CI 0.4–2.8). However, in one trial that permitted the concomitant use of anti-diabetic agents associated with hypoglycemia, hypoglycemic episodes were reported much higher in the subgroup of patients who were taking background insulin or insulin secretagogues (29/87 vs. 12/74 in the treatment and control group, respectively) than those among the patients not on insulin or insulin secretagogues (1/24 vs. 0/28 in the treatment and control group, respectively)^11^. A sensitivity analysis after excluding that study revealed no increased risk of hypoglycemia in the SGLT2i/DPP4i group compared with the PCB/DPP4i group (RR 1.4, 95% CI 0.7–2.9) (Supplementary Figure 8).

All seven studies assessed the risk of UTI and genital infection at the end of the treatment. The definitions for UTI and genital infection were based on the Medical Dictionary for Regulatory Activities (MedDRA) system for 4 out of 7 selected trials^8,9,12,14^, while the rest of the studies did not mention the definition of such conditions. The pooled analysis showed no significant increase in RR of UTI in the SGLT2i/DPP4i group compared with the PCB/DPP4i group, regardless of either a simultaneous or sequential combination (Fig. 4B). However, the risk of genital infection was higher in the SGLT2i/DPP4i group than in the PCB/DPP4i group (RR 2.92, 95% CI 1.29–6.62) (Fig. 4C). The risk of genital infection was increased in the sequential combination subgroup (RR 5.57, 95% CI 2.33–13.333) but not in the simultaneous combination subgroup (RR 1.35, 95% CI 0.55–3.34).

### Exploration of heterogeneity and publication bias

The results of all heterogeneity tests associated with the meta-analyses performed in this study were not statistically significant (p > 0.05). The funnel plot and Egger’s test found no evidence of a small study effect (p = 0.229) (Supplementary Figure 9). It was difficult to assess a risk of publication bias properly because of the small number of included studies.

## Discussion

The present systematic review and meta-analysis revealed the superior glycemic control in SGLT2i/DPP4i than PCB/DPP4i, with greater reduction in HbA1c, FPG, and 2 h PPG, and a higher HbA1c goal achieving rate. The complementary mechanisms of action of SGLT2 inhibitors and DPP4 inhibitors may explain these beneficial effects of the combination. Interestingly, the sequential combination of SGLT2 inhibitors to pre-existing DPP4 inhibitors tended to be more effective than the simultaneous combination of the two drugs. This result might be due to differences in patients’ characteristics at baseline according to the mode of combination. The HbA1c reduction efficacy of an SGLT2 inhibitor was reported to be greater in patients with a higher HbA1c value at baseline^10^. However, as shown in Table 1, the baseline HbA1c appeared to be comparable between the two modes of combination. Another possibility is that the patients in the sequential combination studies, who were inadequately controlled with DPP4 inhibitors, might be more sensitive to SGLT2 inhibitors, which needs further investigation.

The SGLT2i/DPP4i combination showed a significant reduction in body weight compared with PCB/DPP4i in either simultaneous or sequential combination studies. The weight loss effect of SGLT inhibitors is explained by calorie loss through urinary glucose excretion^2,3^. Intriguingly, considering the continuous energy excretion through glycosuria, SGLT2 inhibitors exhibit only a modest amount of weight loss, usually 2–3 kg from baseline^15^. This phenomenon is explained by recent studies showing that weight loss by SGLT2 inhibitors may lead to adaptive increase in appetite and calorie intake^16,17^. The amount of weight loss by SGLT2 inhibitors in combination with DPP4 inhibitors found in our meta-analysis was similar to the previously reported weight-loss effect of SGLT2 inhibitors in general.

The risk of hypoglycemia is one of the major limiting factors when combining multiple agents to reach glycemic goals in patients with type 2 diabetes. It is unlikely that DPP4 inhibitors cause hypoglycemia when used alone or in combination with metformin, due to the glucose-dependent effects on insulin and glucagon secretion^18^. Furthermore, DPP4 inhibitors improve the ability of alpha cells to sense and respond to hypoglycemia^19,20^. SGLT2 inhibitors, when used alone or in combination with metformin, are also associated with reduced risk of hypoglycemia by increasing plasma glucagon concentrations and decreasing plasma insulin concentrations^21,22^. However, in our study, the risk of hypoglycemia increased when SGLT2 inhibitors were sequentially added to pre-existing DPP4 inhibitors, which was probably due to a study that permitted insulin and/or sulfonylureas as background anti-diabetes therapy^11^. Indeed, in the sensitivity analysis excluding the study, the imbalance in the risk of hypoglycemia disappeared.

UTI and genital infection are commonly reported as adverse effects of SGLT2 inhibitors. In a meta-analysis of 58 studies, SGLT2 inhibitors increased the risk of UTI and genital infection compared to placebo (odds ratio [OR] 1.34, 95% CI 1.03–1.74, and OR 3.50, 95% CI 2.46–4.99, respectively)^23^. There was also some concern over increased susceptibility to infection with DPP4 inhibitors. In an early meta-analysis, DPP4 inhibitors were reported to increase the risk of upper respiratory infection and UTI^24^. However, subsequent analysis did not show any increased risk of infection related to the use of DPP4 inhibitors^25^. When we compared SGLT2i/DPP4i and PCB/DPP4i, the risk of UTI and genital infection was not increased regardless of the mode of combination. However, the RR of genital infection increased in the sequential addition of SGLT2 inhibitors to pre-existing DPP4 inhibitors. It is noteworthy that Jabbour *et al*.^12^ reported a very high incidence of genital infection. Although it was unclear why the incidences of genital infection varied widely, it is conceivable that different clinical characteristics of study participants or different reporting rates could be the source of the heterogeneity.

Although the number of studies that reported data on lipid profiles was limited, when compared with the PCB/DPP4i group, the SGLT2i/DPP4i group significantly increased serum HDL-C and LDL-C levels and showed a tendency to decrease serum triglyceride levels. These results were consistent with previous data reported in patients treated with SGLT2 inhibitors^26^. Although the mechanisms by which the SGLT2 inhibitor raises LDL-C levels are not clearly understood, emerging evidences suggest potential mechanisms, such as increased lipoprotein lipase activity, suppressed generation of cholesterol-poor LDL-C, and reduced LDL receptor-mediated LDL clearance by the liver^27^. However, the increased LDL-C does not seem to be connected to a high cardiovascular event risk, which was supported by the EMPA-REG OUTCOME study, exhibiting a 38% reduction in cardiovascular mortality^28^.

There are some limitations in this study. First, the current systematic review and meta-analysis included two different groups of studies in terms of mode of the combination of the two drugs (i.e. a simultaneous and sequential combination). However, the subgroup analyses yielded largely similar results and the I^2^ was not inappropriately high in the pooled analysis for HbA1c (43.1%, p = 0.103). Rather, our analysis showed that SGLT2 inhibitors are efficacious as a combinatorial drug of DPP4 inhibitors in various clinical settings. Second, even though the included studies were well-designed RCTs with a sufficient sample size, the number of included studies was small. Third, the treatment duration was relatively short, which prevented us from concluding the long-term efficacy and safety of the SGLT2i/DPP4i combination therapy. However, to the best of our knowledge, the present meta-analysis is the first to evaluate the efficacy and safety of the combination of an SGLT2 inhibitor and a DPP4 inhibitor in patients with inadequately controlled type 2 diabetes.

In conclusion, the SGLT2 inhibitor and DPP4 inhibitor combination therapy improves glycemic control and reduces body weight without increasing the risk of hypoglycemia and UTI in patients with inadequately controlled type 2 diabetes. Because SGLT2 inhibitors and DPP4 inhibitors are newer and more expensive drugs, the long-term efficacy and safety and the cost-effectiveness of the combination need to be examined.

## Electronic supplementary material

Supplementary information
