# Supplementary material for: Combination of sodium-glucose cotransporter 2 inhibitor and dipeptidyl peptidase-4 inhibitor in type 2 diabetes: a systematic review with meta-analysis
Source: Sci Rep. 2018 Mar 13;8:4466. doi: 10.1038/s41598-018-22658-2 (PMC5849757; doi:10.1038/s41598-018-22658-2)

Combination of sodium-glucose cotransporter 2 inhibitor and dipeptidyl peptidase-4 inhibitor in type 2 diabetes: a systematic review with meta-analysis

Short title: Combination of SGLT2 Inhibitors and DPP4 Inhibitors

Se Hee Min[1]*, Jeong-Hwa Yoon[2]*, Sun Joon Moon[1], Seokyung Hahn[3]**, Young Min Cho[1]**

[1]Division of Endocrinology and Metabolism, Department of Internal Medicine, Seoul National University College of Medicine, Seoul, South Korea

[2]Interdisciplinary Program in Medical Informatics, Seoul National University College of Medicine, Seoul, South Korea

[3]Department of Medicine, Seoul National University College of Medicine, Seoul, South Korea

* S.H.M. and J.-H.Y. contributed equally to this work

** S.H. and Y.M.C. are co-corresponding authors

Correspondence:

Young Min Cho, MD, PhD Co-corresponding author

Department of Internal Medicine, Seoul National University College of Medicine

101 Daehak-ro, Jongno-gu, Seoul 03080, Korea

Tel: +82-2-2072-1965

Fax: +82-2-762-9662

E-mail: ymchomd@snu.ac.kr

Seokyung Hahn, PhD, Co-corresponding author

Department of Medicine, Seoul National University College of Medicine

103 Daehak-ro, Jongno-gu, Seoul 03080, Korea

Tel: +82-2-740-8911

Fax: +82-2-743-8361

E-mail: hahns@snu.ac.kr

Supplementary Text S1. Search term strategy

MEDLINE

#1 dipeptidyl peptidase iv inhibitors[Title/Abstract] OR dipeptidyl peptidase iv inhibitors[Title/Abstract] OR dipeptidyl peptidase-4 inhibitor[Title/Abstract] OR dipeptidyl peptidase 4 inhibitor[Title/Abstract] OR DPP4[Title/Abstract] OR DPP-4[Title/Abstract] OR Sitagliptin[Title/Abstract] OR MK-0431[Title/Abstract] OR Vildagliptin[Title/Abstract] OR LAF237[Title/Abstract] OR Dutogliptin[Title/Abstract] OR PHX1149[Title/Abstract] OR Saxagliptin[Title/Abstract] OR BMS-477118[Title/Abstract] OR Linagliptin[Title/Abstract] OR BI1356[Title/Abstract] OR Alogliptin[Title/Abstract] OR SYR-322[Title/Abstract] OR Gemigliptin[Title/Abstract] OR LC15-0444[Title/Abstract] OR Tenegliptin[Title/Abstract] OR MP-513[Title/Abstract] OR Anagliptin[Title/Abstract] OR SK-0403[Title/Abstract] OR Gosogliptin[Title/Abstract] OR PF-734200[Title/Abstract] OR Evogliptin[Title/Abstract] OR DA-1229[Title/Abstract]

#2 sodium glucose transporter 2[Title/Abstract] OR sodium-glucose transporter 2[Title/Abstract] OR SGLT2[Title/Abstract] OR SGLT-2[Title/Abstract] OR Dapagliflozin[Title/Abstract] OR BMS-512148[Title/Abstract] OR Canagliflozin[Title/Abstract] OR TA-7284[Title/Abstract] OR Empagliflozin[Title/Abstract] OR BI-10773[Title/Abstract] OR Ipragliflozin[Title/Abstract] OR ASP1941[Title/Abstract] OR Luseogliflozin[Title/Abstract] OR TS-071[Title/Abstract] OR Tofogliflozin[Title/Abstract] OR CSG452[Title/Abstract] OR R7201[Title/Abstract] OR RG7201[Title/Abstract] OR Ertugliflozin[Title/Abstract] OR MK-8835[Title/Abstract] OR PF-04971729[Title/Abstract]

#3 #1 and #2

#4 randomized controlled trial [pt]

#5 controlled clinical trial [pt]

#6 randomized [tiab]

#7 placebo [tiab]

#8 clinical trials as topic [mesh: noexp]

#9 randomly [tiab]

#10 trial [ti]

#11 #4 OR #5 OR #6 OR #7 OR #8 OR #9 OR #10

#12 animals [mh] NOT humans [mh]

#13 #11 not #12

#14 #3 and #13

EMBASE

#1 ‘dipeptidyl peptidase iv inhibitors’:ab,ti OR ‘dipeptidyl peptidase iv inhibitors’:ab,ti OR ‘dipeptidyl peptidase-4 inhibitor’:ab,ti OR ‘dipeptidyl peptidase 4 inhibitor’:ab,ti OR DPP4:ab,ti OR DPP-4:ab,ti OR Sitagliptin:ab,ti OR MK-0431:ab,ti OR Vildagliptin:ab,ti OR LAF237:ab,ti OR Dutogliptin:ab,ti OR PHX1149:ab,ti OR Saxagliptin:ab,ti OR BMS-477118:ab,ti OR Linagliptin:ab,ti OR BI1356:ab,ti OR Alogliptin:ab,ti OR SYR-322:ab,ti OR Gemigliptin:ab,ti OR LC15-0444:ab,ti OR Tenegliptin:ab,ti OR MP-513:ab,ti OR Anagliptin:ab,ti OR SK-0403:ab,ti OR Gosogliptin:ab,ti OR PF-734200:ab,ti OR Evogliptin:ab,ti OR ‘DA-1229’:ab,ti

#2 ‘sodium glucose transporter 2’:ab,ti OR ‘sodium-glucose transporter 2’:ab,ti OR SGLT2:ab,ti OR SGLT-2:ab,ti OR Dapagliflozin:ab,ti OR BMS-512148:ab,ti OR Canagliflozin:ab,ti OR TA-7284:ab,ti OR Empagliflozin:ab,ti OR BI-10773:ab,ti OR Ipragliflozin:ab,ti OR ASP1941:ab,ti OR Luseogliflozin:ab,ti OR TS-071:ab,ti OR Tofogliflozin:ab,ti OR CSG452:ab,ti OR R7201:ab,ti OR RG7201:ab,ti OR Ertugliflozin:ab,ti OR MK-8835:ab,ti OR PF-04971729:ab,ti

#3 #1 and #2

#4 'crossover procedure':de OR 'double-blind procedure':de OR 'randomized controlled trial':de OR 'single-blind procedure':de OR random*:de,ab,ti OR factorial*:de,ab,ti OR crossover*:de,ab,ti OR (cross NEXT/1 over*):de,ab,ti OR placebo*:de,ab,ti OR (doubl* NEAR/1 blind*):de,ab,ti OR (singl* NEAR/1 blind*):de,ab,ti OR assign*:de,ab,ti OR allocat*:de,ab,ti OR volunteer*:de,ab,ti

#5 #3 and #4

The Cochrane Library

#1 ‘dipeptidyl peptidase iv inhibitors’:ab,ti OR ‘dipeptidyl peptidase iv inhibitors’:ab,ti OR ‘dipeptidyl peptidase-4 inhibitor’:ab,ti OR ‘dipeptidyl peptidase 4 inhibitor’:ab,ti OR DPP4:ab,ti OR DPP-4:ab,ti OR Sitagliptin:ab,ti OR MK-0431:ab,ti OR Vildagliptin:ab,ti OR LAF237:ab,ti OR Dutogliptin:ab,ti OR PHX1149:ab,ti OR Saxagliptin:ab,ti OR BMS-477118:ab,ti OR Linagliptin:ab,ti OR BI1356:ab,ti OR Alogliptin:ab,ti OR SYR-322:ab,ti OR Gemigliptin:ab,ti OR LC15-0444:ab,ti OR Tenegliptin:ab,ti OR MP-513:ab,ti OR Anagliptin:ab,ti OR SK-0403:ab,ti OR Gosogliptin:ab,ti OR PF-734200:ab,ti OR Evogliptin:ab,ti OR ‘DA-1229’:ab,ti

#2 ‘sodium glucose transporter 2’:ab,ti OR ‘sodium-glucose transporter 2’:ab,ti OR SGLT2:ab,ti OR SGLT-2:ab,ti OR Dapagliflozin:ab,ti OR BMS-512148:ab,ti OR Canagliflozin:ab,ti OR TA-7284:ab,ti OR Empagliflozin:ab,ti OR BI-10773:ab,ti OR Ipragliflozin:ab,ti OR ASP1941:ab,ti OR Luseogliflozin:ab,ti OR TS-071:ab,ti OR Tofogliflozin:ab,ti OR CSG452:ab,ti OR R7201:ab,ti OR RG7201:ab,ti OR Ertugliflozin:ab,ti OR MK-8835:ab,ti OR PF-04971729:ab,ti

#3 #1 and #2

Supplementary Figure S1. Risk of bias assessment.

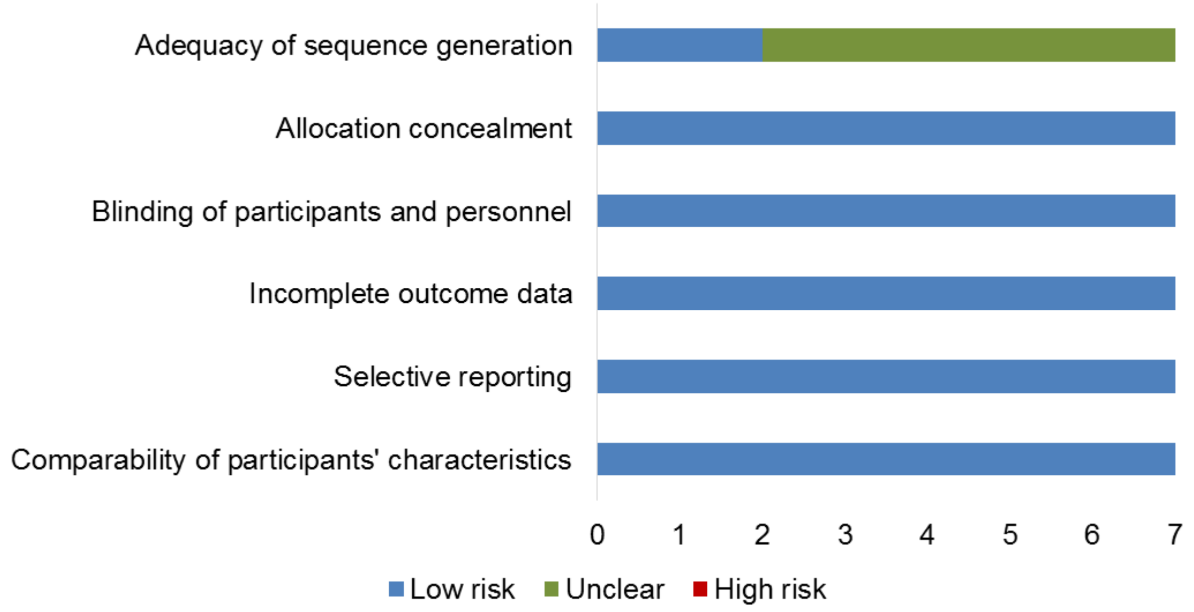

Supplementary Figure S2. Weighted mean difference in change in 2-h postprandial glucose from baseline.

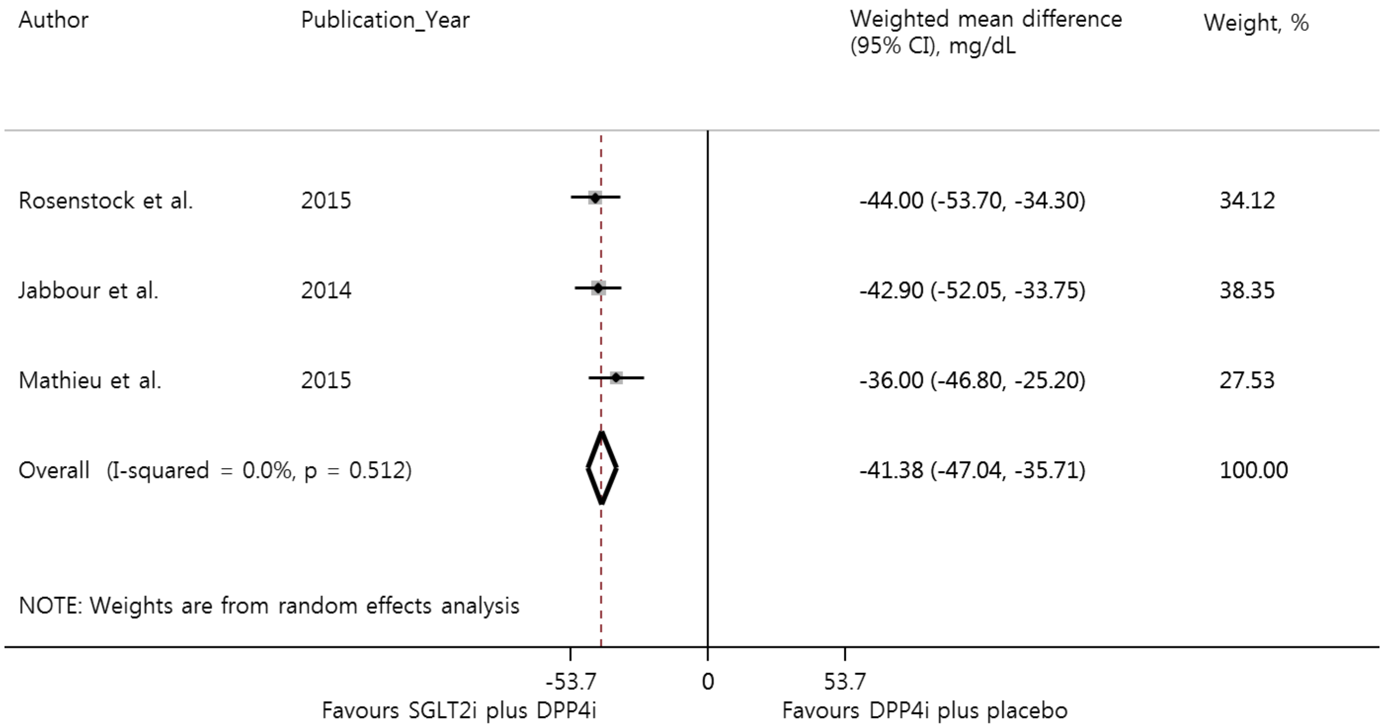

The weighted mean difference in change in 2-h postprandial glucose (mg/dL) from baseline with SGLT2 inhibitor plus DPP4 inhibitor versus placebo plus DPP4 inhibitor is shown. The squares represent an individual study's effects, and the size of squares reflects the study's weight with the horizontal lines extending from the symbols representing 95% CIs. The diamonds indicate the pooled estimates.

CIs = confidence intervals; PCB/DPP4i = placebo plus DPP4 inhibitor; SGLT2i/DPP4i = SGLT2 inhibitor plus DPP4 inhibitor.

Supplementary Figure S3. Weighted mean difference in change in systolic blood pressure from baseline.

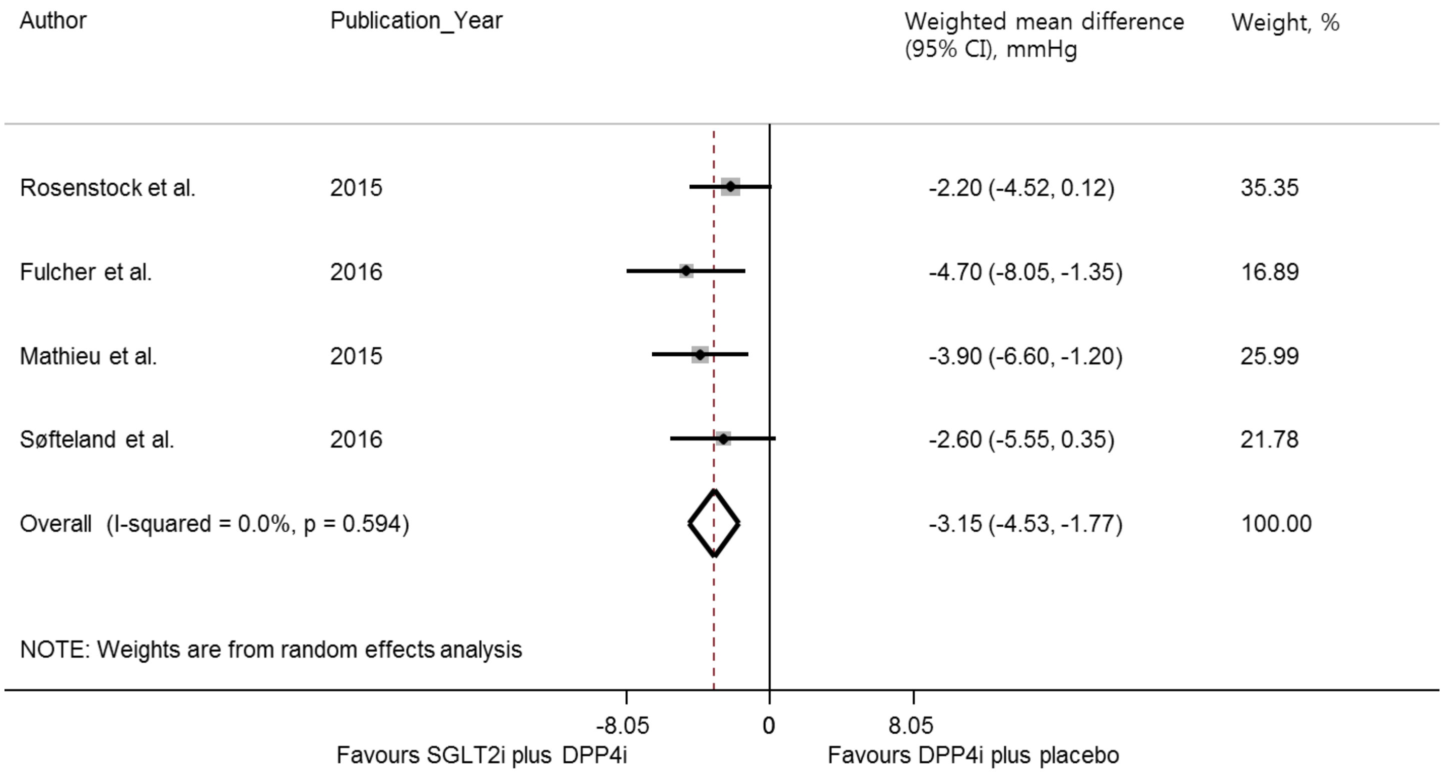

The weighted mean difference in change in systolic blood pressure (mmHg) from baseline with SGLT2 inhibitor plus DPP4 inhibitor versus placebo plus DPP4 inhibitor is shown. The squares represent an individual study's effects, and the size of squares reflects the study's weight with the horizontal lines extending from the symbols representing 95% CIs. The diamonds indicate the pooled estimates.

CIs = confidence intervals; PCB/DPP4i = placebo plus DPP4 inhibitor; SGLT2i/DPP4i = SGLT2 inhibitor plus DPP4 inhibitor.

Supplementary Figure S4. Weighted mean difference in the percentage change in total cholesterol from baseline.

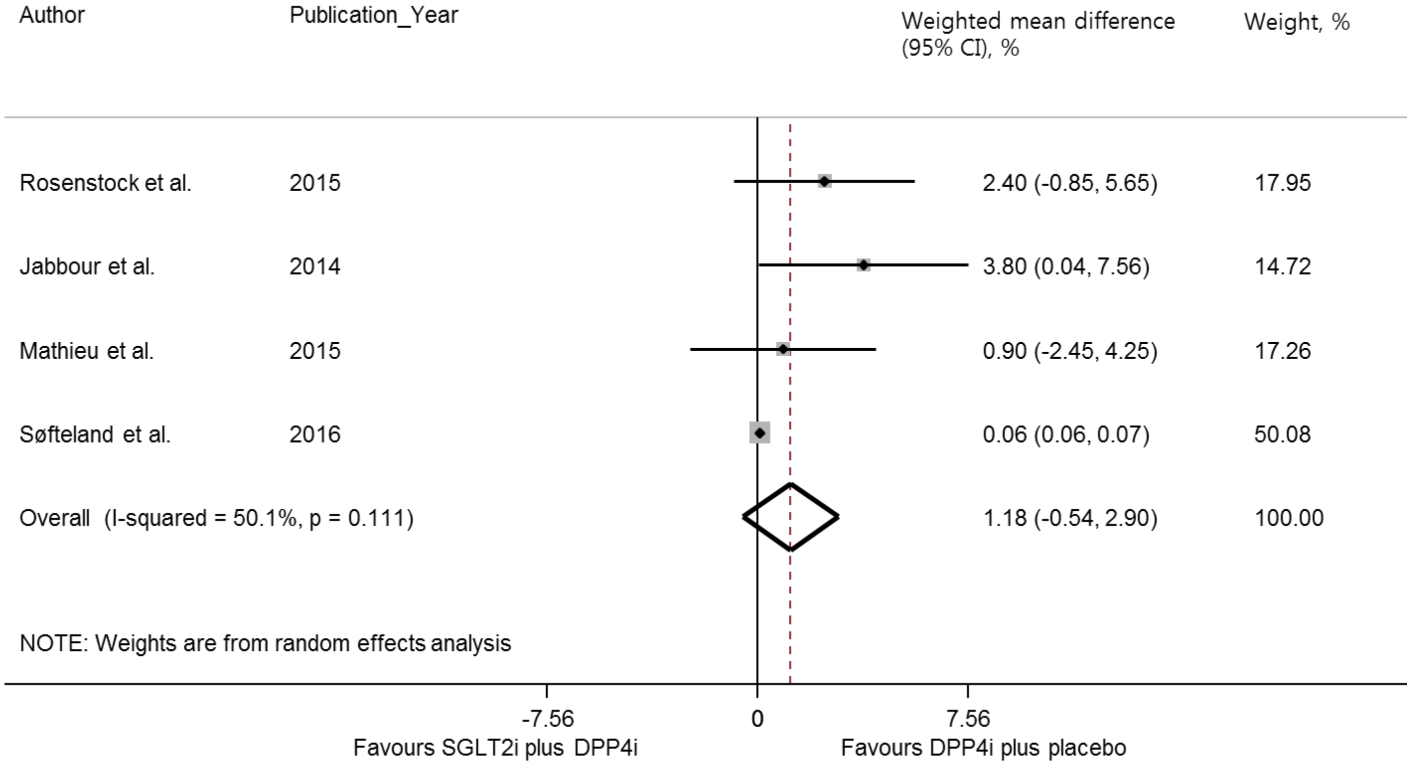

The weighted mean difference in the percentage change in total cholesterol (%) from baseline with SGLT2 inhibitor plus DPP4 inhibitor versus placebo plus DPP4 inhibitor is shown. The squares represent an individual study's effects, and the size of squares reflects the study's weight with the horizontal lines extending from the symbols representing 95% CIs. The diamonds indicate the pooled estimates.

CIs = confidence intervals; PCB/DPP4i = placebo plus DPP4 inhibitor; SGLT2i/DPP4i = SGLT2 inhibitor plus DPP4 inhibitor.

Supplementary Figure S5. Weighted mean difference in the percentage change in triglyceride from baseline.

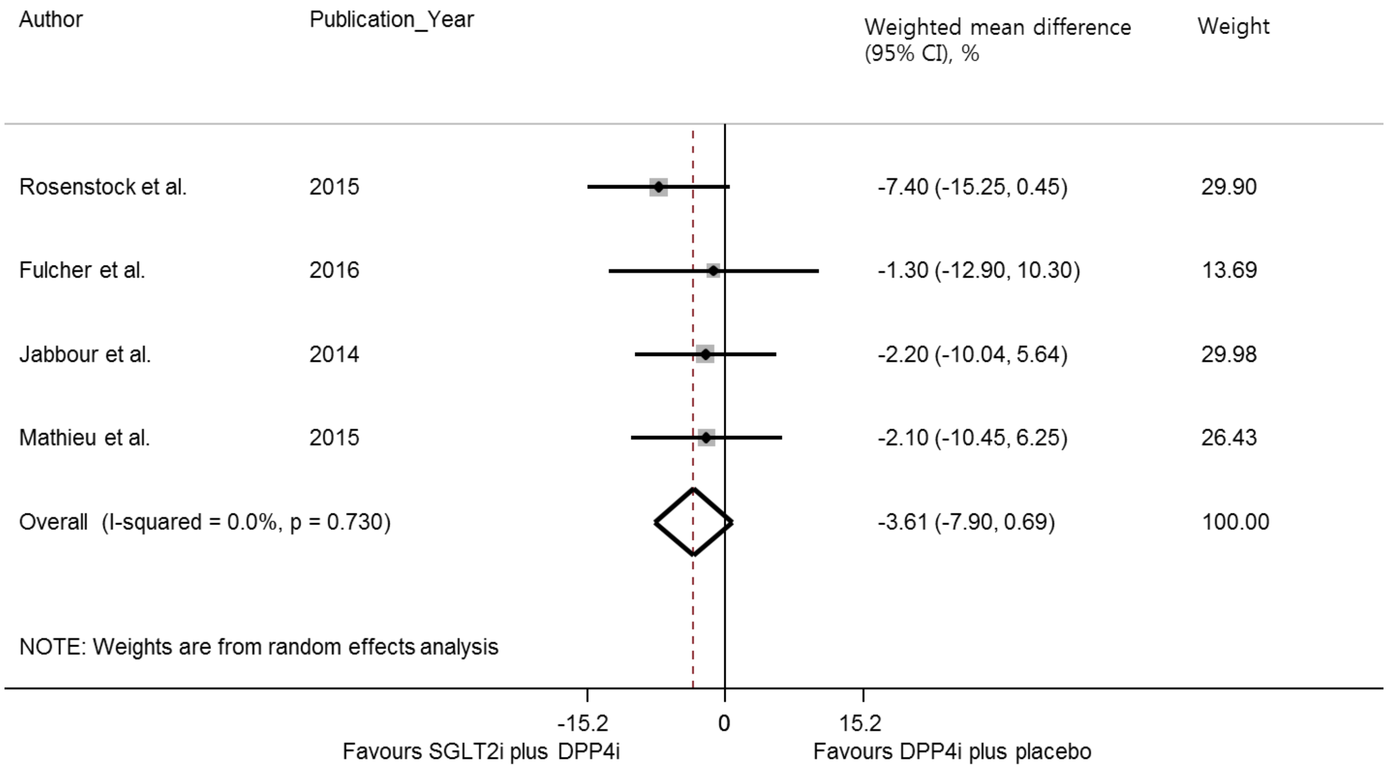

The weighted mean difference in the percentage change in triglyceride (%) from baseline with SGLT2 inhibitor plus DPP4 inhibitor versus placebo plus DPP4 inhibitor is shown. The squares represent an individual study's effects, and the size of squares reflects the study's weight with the horizontal lines extending from the symbols representing 95% CIs. The diamonds indicate the pooled estimates.

CIs = confidence intervals; PCB/DPP4i = placebo plus DPP4 inhibitor; SGLT2i/DPP4i = SGLT2 inhibitor plus DPP4 inhibitor.

Supplementary Figure S6. Weighted mean difference in the percentage change in LDL-C from baseline.

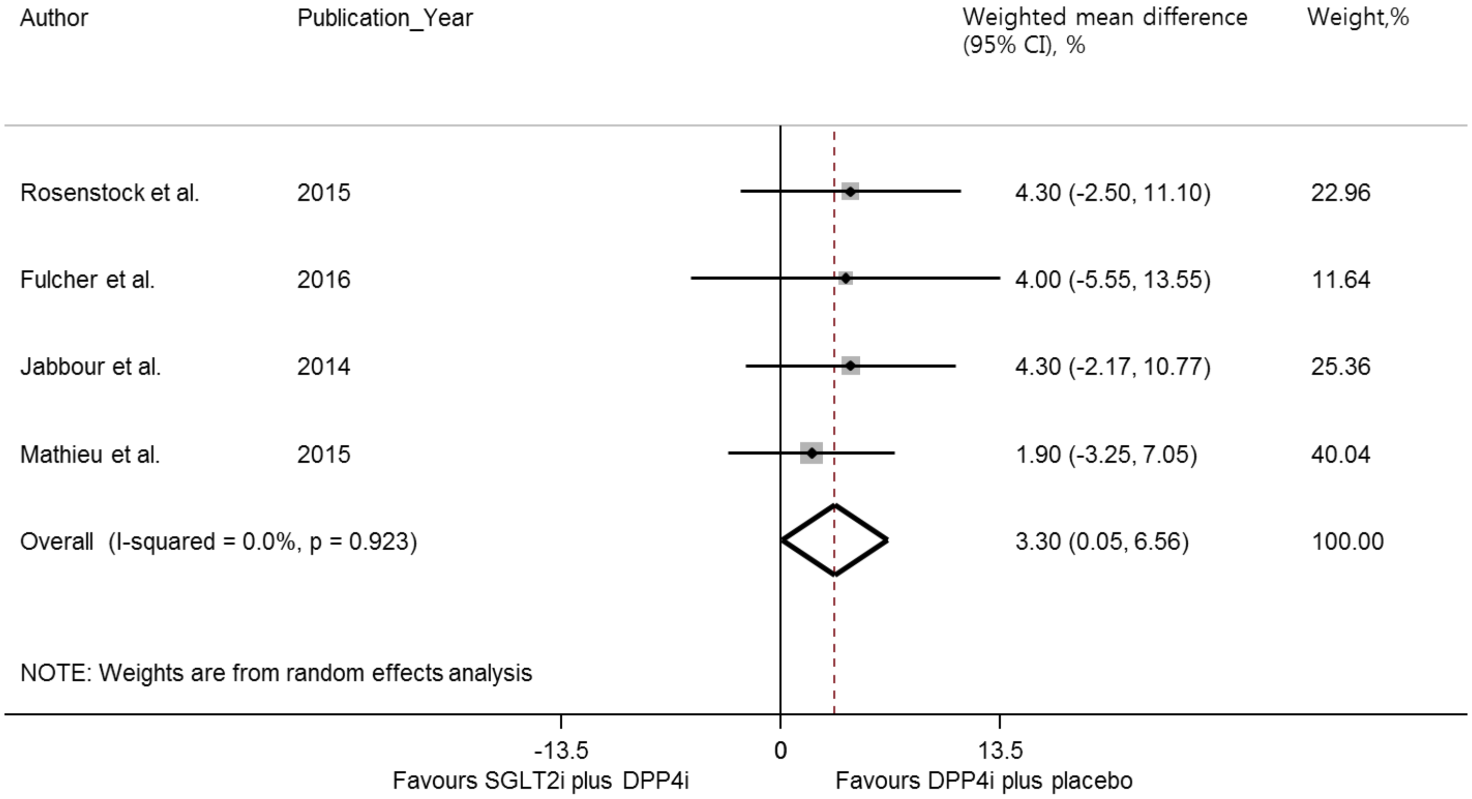

The weighted mean difference in the percentage change in LDL-C (%) from baseline with SGLT2 inhibitor plus DPP4 inhibitor versus placebo plus DPP4 inhibitor is shown. The squares represent an individual study's effects, and the size of squares reflects the study's weight with the horizontal lines extending from the symbols representing 95% CIs. The diamonds indicate the pooled estimates.

CIs = confidence intervals; PCB/DPP4i = placebo plus DPP4 inhibitor; SGLT2i/DPP4i = SGLT2 inhibitor plus DPP4 inhibitor.

Supplementary Figure S7. Weighted mean difference in the percentage change in HDL-C from baseline.

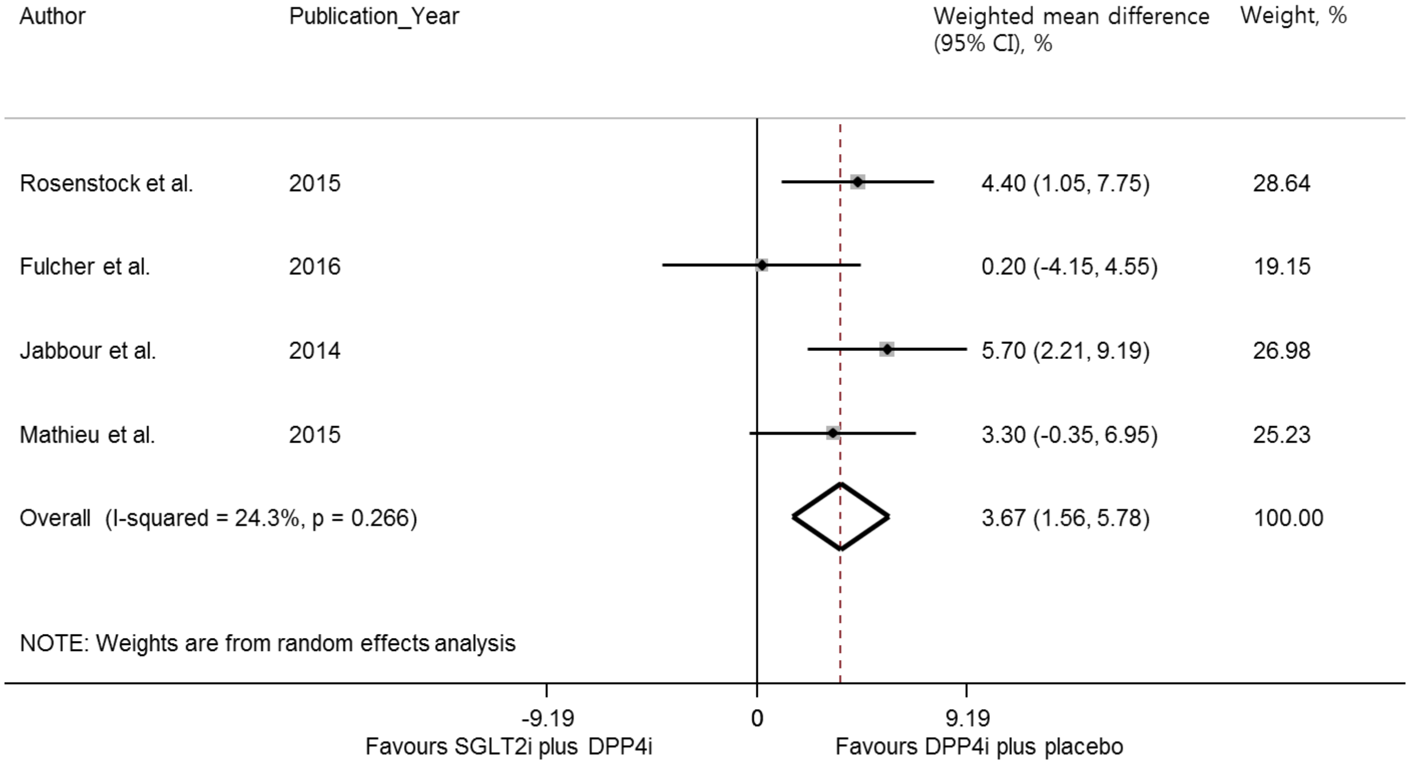

The weighted mean difference in the percentage change in HDL-C (%) from baseline with SGLT2 inhibitor plus DPP4 inhibitor versus placebo plus DPP4 inhibitor analyzed isshown. The squares represent an individual study's effects, and the size of squares reflects the study's weight with the horizontal lines extending from the symbols representing 95% CIs. The diamonds indicate the pooled estimates.

CIs = confidence intervals; PCB/DPP4i = placebo plus DPP4 inhibitor; SGLT2i/DPP4i = SGLT2 inhibitor plus DPP4 inhibitor.

Supplementary Table S1. Definitions of hypoglycemia in the studies used for the meta-analyses.

Source	Definition of hypoglycemia	Definition of major or severe hypoglycemia
DeFronzo et al, 2015	Plasma glucose ≤70 mg/dl and/or requiring assistance	
Lewin et al, 2015	Plasma glucose ≤70 mg/dl and/or requiring assistance	
Rosenstock 2015	Minor: symptomatic or asymptomatic with plasma glucose concentration <63 mg/dl, regardless of need for external assistance. Major: See the right column.	Requiring the assistance of others to treat their hypoglycemic symptoms, with or without plasma glucose concentration < 54 mg/dl, and prompt recovery after glucose or glucagon administration.
Fulcher et al, 2016	Biochemically documented [≤3.9 mmol/l (70 mg/dl)]. Severe: See the right column.	Requiring the assistance of others, or loss of consciousness or seizure.
Jabbour et al. 2014	Not available	
Mathieu et al. 2015	Minor: symptomatic or asymptomatic with plasma glucose concentration <63 mg/dl, regardless of need for external assistance. Major: See the right column.	Requiring the assistance of others to treat their hypoglycemic symptoms, with or without plasma glucose concentration < 54 mg/dl, and prompt recovery after glucose or glucagon administration.
Søfteland et al. 2016	Plasma glucose ≤3.9 mmol/l and/or requiring assistance	

Supplementary Figure S8. Sensitivity analysis for the meta-analysis of relative risk of hypoglycemia.

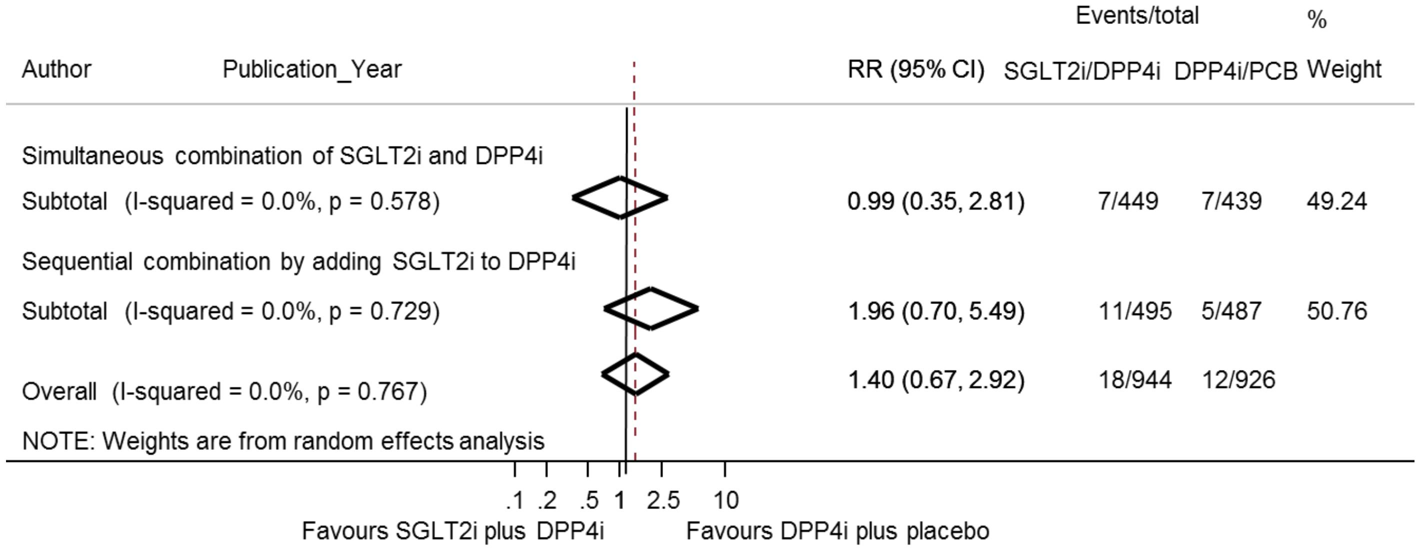

The relative risk of hypoglycemia with SGLT2 inhibitor plus DPP4 inhibitor compared with placebo plus DPP4 inhibitor is shown. In this sensitivity analysis, one trial that permitted insulin or insulin secretagogues was excluded. The diamonds indicate the pooled estimate.

CIs = confidence intervals; PCB/DPP4i = placebo plus DPP4 inhibitor; SGLT2i/DPP4i = SGLT2 inhibitor plus DPP4 inhibitor.

Supplementary Figure S9. Funnel plot and Egger's test for the primary outcome**.**

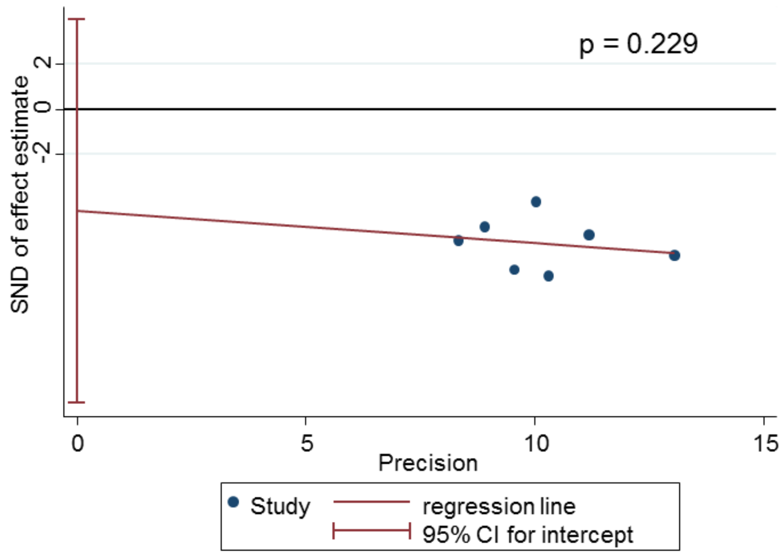

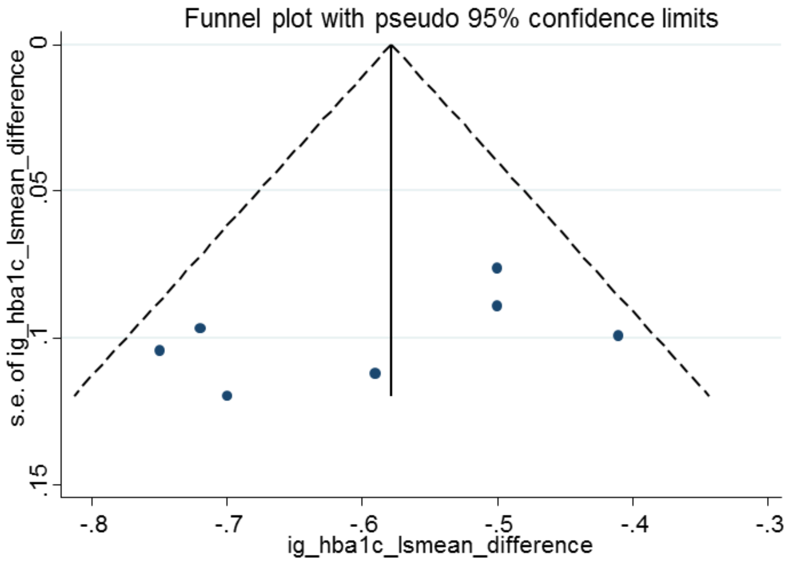

Supplement: Supplementary file 1 — Supplementary information [file 41598_2018_22658_MOESM1_ESM.pdf]
